# Looks like smoking, is it smoking?: Children’s perceptions of cigarette-like nicotine delivery systems, smoking and cessation

**DOI:** 10.1186/1477-7517-10-30

**Published:** 2013-11-18

**Authors:** Julienne Faletau, Marewa Glover, Vili Nosa, Fiona Pienaar

**Affiliations:** 1 Centre for Tobacco Control Research, School of Population Health, University of Auckland, Private 92019, Auckland 1142, New Zealand; 2 Pacific Health, School of Population Health, University of Auckland, Private Bag 92019, Auckland 1142, New Zealand; 3 Faculty of Education, The University of Cambridge, Cambridge, England

**Keywords:** Smoking, Nicotine, E-cigarette, Children

## Abstract

**Background:**

Alternative cigarette-like nicotine delivery systems have been met with diverse opinions. One concern has been for the effect on children. We investigate whether children can differentiate tobacco cigarette smoking from use of a nicotine inhaler and electronic cigarette. Their opinions on these devices was also of interest.

**Methods:**

Two structured focus groups and twelve individual interviews were conducted with twenty Māori and Pacific children (6–10 years old) in low socioeconomic areas in Auckland, New Zealand. Children viewed short video clips on an iPad that demonstrated an actor smoking a tobacco cigarette, sucking a lollipop or using an electronic cigarette or a nicotine inhaler.

**Results:**

Children did not recognise the inhaler or electronic cigarette. Some children did however notice anomalies in the ‘smoking’ behaviour. Once told about the products the children were mostly positive about the potential of the inhaler and electronic cigarette to assist smokers to quit. Negative perceptions were expressed, including views about the ill health effects associated with continued nicotine intake and the smoker’s inability to quit.

**Conclusions:**

In a context unfamiliar with electronic cigarettes or nicotine inhalers, such as New Zealand, children may misperceive use of these products as smoking. Once these products are more common and the purpose of them is known, seeing people use them should normalise quitting behaviour, something the children were very supportive of.

## Introduction

Smoking is an addiction most often started in childhood. The earlier a child begins to smoke, the less likely they are to quit smoking as an adult, and the more likely they are to die prematurely from a smoking-related disease (Gordon, Mackay, & Rehfuess, [1]; Health Sponsorship Council., [2]). Despite the well-known health risks associated with smoking, 7.7% of 14–15 year olds in New Zealand (NZ) are regular tobacco smokers ([3]; Health Sponsorship Council., [2]). Seventeen per cent of youth who report having ever smoked a cigarette did so before the age of 10 years and the proportion is higher among Māori and Pacific Island people (Health Sponsorship Council., [2]). The prevalence of smoking is higher in socio-economically deprived areas in NZ and health outcomes related to tobacco disease are high in lower socioeconomic groups, where Māori and Pacific people are over-represented [4]. Smoking prevalence is much higher among Māori and Pacific people (45% and 33%) versus Pakeha/European.

Reducing initiation to smoke by denormalising tobacco use is a key strategy of NZ’s tobacco control programme which is aiming for NZ to be smokefree by 2025, defined as a smoking prevalence of 5% or below (Health Sponsorship Council., [2]). Underpinned by social learning theory [5] it is believed that children’s attitudes and values toward smoking are formed in part from observing others modelling the behaviour [5]. Even unhealthy behaviours when displayed by role models are accepted by children and mimicked. Initiation and maintenance of smoking behavior are predominately influenced by social models, namely parents, siblings and peers, as well as influences such as tobacco smoking imagery in the media and tobacco-like confectionary ([6]–[8]; Ministry of Health., [9]). Parental smoking behaviour and peer influences have been found to be key determinants of smoking in NZ [10]. Thus, NZ’s denormalisation strategy aims to reduce children’s exposure to adults role-modelling smoking.

Most of the first generation of electronic cigarettes replicate the appearance and many of the behavioural aspects of tobacco smoking [11]–[13]. Some newer models diverge from this look and instead have been likened to devices used for smoking cannabis, (for example, bongs) in both look and behavioural use aspects [14]. A proven nicotine replacement device is the nicotine inhaler [12,15,16]. The nicotine inhaler is a small plastic device that is held between thumb and forefinger to the mouth much like a cigarette can be. The nicotine inhaler and electronic cigarette can provide the smoker with nicotine, as well as the rituals associated with tobacco smoking behaviour, such as the hand-to-mouth action and the appearance of a smoke-like mist or physical sensation of inhaling [12]. Tobacco control advocates against the electronic cigarette are concerned that use of electronic cigarettes model smoking-like behaviour [17] and will thus continue to ‘normalise smoking for children’ [18]. Under NZ’s Smoke-free Environments Act (1990) the electronic cigarette is categorized as a tobacco product, however arguments arise that electronic cigarettes could undermine smokefree environments laws that prohibit smoking inside workplaces, restaurants and bars, on school grounds, in public transport and in hospitals ([19,20],Laugesen [21]).

Children potentially develop vulnerability to smoke at a very young age. Several studies have found young children with susceptible cognitions to smoke. Schuck et al. found a positive association between the numbers of smokers among parents, siblings and peers and children’s perceptions of the advantages of smoking [22]. Children of parents who smoke perceived casual smoking to be safer and then reported wanting to smoke in response to smoking-related cues more than children of non-smoking parents [22]. When asked to pretend that they were grown-ups having dinner, 30% of young children who reported having parents who smoke, pretended to smoke after having dinner [23]. De Leeuw and colleagues found that even in the absence of the role model, participants knew in detail how to light up a cigarette and smoke it [23]. Candy cigarettes have been linked to the development of children’s attitudes towards smoking as acceptable, favourable and a normative behaviour in a study with preschool and primary school aged children [24].

The belief in social learning theory is a significant driver of the fear expressed towards cigarette-like nicotine delivery devices. There is however limited literature on children’s perceptions of such devices and their role, if any, in child smoking uptake. Research is needed to determine if electronic cigarettes and nicotine inhalers increase the risk of child uptake of smoking. Our study tested if children (aged 6–10 years) could discern the differences between a stranger at a distance smoking a cigarette versus using an electronic cigarette or nicotine inhaler or sucking a lollipop. The attitudes of children towards these devices was also of interest.

## Methods

### Study design

A qualitative exploratory design using structured focus groups and individual interviews.

### Participants and procedure

Study participants were recruited from two low socioeconomic primary schools located in East and South Auckland, NZ. Participating schools were chosen for their large proportions of Māori and Pacific ethnicities. Participants were chosen if their parents were smokers or if teachers knew that the student had a respiratory condition. An envelope containing a cover letter, information sheet and consent form was provided to teachers to send home to parents of selected children. Only children whose parents or guardians granted consent were included. Parents were invited to provide contact details if they wanted to receive a summary report of the study findings. Twenty Māori, Tongan, Samoan, Cook Island and Niuean children aged 6–10 years were recruited. Twelve were interviewed on their own and the rest participated in two focus groups (with five in the 10 year old’s group and 3 in the eight year old’s group).

## Materials

Eight 30 second video clips of scenarios showing ‘smoking’ behaviour were produced: four in a park and four in a bus stop. The park scenario showed a group of children playing with a ball and an actor in the background using an electronic cigarette, smoking a tobacco cigarette, using a nicotine inhaler or sucking a lollipop. The actor was shown removing an electronic cigarette from her pocket, using it as the children’s ball then rolls towards her. She returns the electronic cigarette to her pocket and gives the ball back to a child. The scene at the bus stop shows a young man with a younger boy waiting to catch a bus and near them in the bus stop the actor smokes or uses the other products. A script was used to ensure consistency of the behaviours to be portrayed across all video clips, (see Table 1). An iPad was used to show the video clips.

**Table 1:** Visual cues script

**Visual Cues/Product**	**Electronic cigarette**	**Nicotine inhaler**	**Tobacco cigarette**	**Lollipop**
**Takes product from pocket or bag**	✔	✔	✔	✔
**Takes product from cigarette box**	✘	✘	✔	✘
**Lights product**	✘	✘	✔	✘
**Hides product behind back**	✘	✘	✔	✘
**Leaves product in mouth**	✘	✘	✘	✔
**Mist or smoke can be seen**	✔	✘	✔	✘
**Puts product back in pocket**	✔	✔	✘	✘

### Interview process

Interviews of 30–40 minutes duration were conducted in August 2011. Focus groups were age-specific as recommended in the literature to facilitate conversation to flow without the children feeling overpowered [25]. Interviews were conducted in a private room at school during regular school hours. Interviews began by building rapport through the use of positive non-verbal cues (e.g. smiling), introductions and allowing participants to play a game on the iPad. Participants were then informed that their parents/guardian had given the interviewer permission to talk to them. The participant information booklet (a simplified participant information sheet) was read aloud by the interviewer and verbal assent to participate was sought from the children.

The children were then shown either the park or bus stop set of video clips depending on a preset schedule to ensure an even mix of scenarios was shown across age groups and in varied order. They were then asked “What do you remember seeing in the video clips?” They were prompted to tell the interviewer what they thought was happening in the video clips. Once the participants provided their answers, the interviewer replayed the tobacco cigarette and electronic cigarette videos and then asked “Did you notice a difference in these video clips?” To prompt for more discussion the interviewer asked. ‘Have you heard of an electronic cigarette?’ If the participants answered ‘No’, the interviewer explained that the electronic cigarette looks like a tobacco cigarette but is electronically charged. Furthermore, smokers use them to help them quit smoking because they do not contain as many chemicals as the tobacco cigarette. The nicotine inhaler and lollipop video clips were then replayed and participants were asked if they noticed a difference. The interviewer then asked ‘Have you heard of a nicotine inhaler?’ If the participants answered, ‘No’, the interviewer explained that the nicotine inhaler is a plastic device that once inhaled, nicotine flows into the person’s body but no smoke comes out of it. It was also explained that the nicotine inhaler is a cessation product that can be used to help smokers quit smoking.

### Data analysis

Audio recordings of interviews were transcribed and checked against the interviewer’s written notes. The transcripts were read and text identified as usable data was extracted, divided into ‘units of analysis’ and grouped according to key themes, based upon the interview schedule. An inductive approach was used to categorise the data allowing for research findings to emerge from the data, despite its deductive beginnings [26].

## Results

### Initial reaction to the video clips

Upon first viewing of the video clips showing use of the electronic cigarette or inhaler and without priming as to the content, several children noticed anomalies in the ‘smoking’ behavior and some did not. One participant who did not notice any difference between use of the products said *“She keeps on smoking, over and over”* (Female, Tongan, 10 years). Those who noticed anomalies in the ‘smokers’ behavior thought it was strange that the actor did not light the ‘cigarette’, as one participant explained it, *“he’s pretending that he’s smoking”* (Male, Tongan, 8 years). Several participants noticed that the actor placed the electronic cigarette or nicotine inhaler into their pocket without extinguishing it which they noted as strange: *“Kind of stupid to put in your pocket… you might get a rip and ash in your pocket”* (Female, Tongan, 10 years).

### Children’s perceptions about the electronic cigarette and nicotine inhaler

Once the children had been told about the electronic cigarette, they were interested in it and thought it was ‘cool’ that it was rechargeable, *“Cool… cause you can charge it like a phone and it lasts longer”* (Male, Tongan, 9 years). Participants viewed the electronic cigarette as an imitation cigarette that could usefully help people stop smoking. *“I think it’s a good way stopping people smoking… they don’t die… their children don’t get asthma and get sick… it’s a way by saving your family’s life and saving your life too”* (Female, Māori, 8 years). One participant noted that the electronic cigarette is bad, because the product still allows the smoker to smoke, despite its function as a cessation aid. *“Bad because they’re still gonna smoke”* (Male, Tongan, 9 years).

When participants were told about the function of the nicotine inhaler, one participant thought if the actor continued using the nicotine inhaler, they might get used to it and not quit. Participants responded by saying, *“…they might get used to it and keep on smoking and stop for a while and then keep on smoking”* (Female, Māori, 8 years). Another participant thought the nicotine inhaler was innovative as it did not need to be lit. *“Cool… cause you don’t have to get a lighter…”* (Male, Tongan, 6 years).

### Children’s perception about the ill-health effects of smoking

The focus group of eight year olds was most knowledgeable about the health effects of smoking which they credited to the graphic pictures on their parent’s cigarette packet. *“Her lungs are going to get bad”* (Male, Tongan, 10 years); *“You’ll get cancer”* (Male, Samoan, 8 years); *“You’ll get rotten teeth”* (Female, Cook Island/Māori, 8 years); *“It makes you ugly”* (Male, Samoan, 8 years). Several participants spoke about secondhand smoke exposure: *“Blow out smoke, makes other peoples lungs go bad too”* (Male, Tongan, 8 years); asthma: *“I’m not gonna smoke cause I’m asthmatic”* (Tongan, Male, 9 years) and the ill health effects on the organs inside and features outside of the body: *“My mum was still carrying my brother and I think she was smoking cause my brother he came out and he had something wrong with his lungs and he got older and he became asthmatic”* (Niuean, Female, 10 years); *“My nena she stop smoking cause she has cancer”* (Female, Cook Island/Maori, 8 years).

### Children’s judgments about smoking and quitting

The children thought it good to stop smoking also because of the prohibitive cost of smoking: *“The smoke that uses the fire, you waste money for it”* (Male, Tongan, 10 years). There was mention that minors should not smoke: *“She shouldn’t smoke cause of her age, she’s young”* (Female, Tongan, 10 years) and of the impact of role modelling behavior of adults: *“You should not smoke cause kids might grow up and smoke too”* (Male, Tongan, 10 years). One child thought parents could better spend their time on their children: *“I think she should quit smoking and have time to play with the people she loves”* (Female, Niuean, 10 years).

### Children’s views about smokefree areas

The children felt there should be smokefree policies in public places such as parks and bus stops especially in areas where children play. *“There should be a policy that there’s no smoking in children’s play area”* (Female, Niuean, 10 years). *“There should be a smokefree sign”* (Male, Tongan, 10 years).

### Family and peer smoking

Children across all interviews readily talked about family and peers smoking. They especially expressed concern about other children smoking: *“This boy in our class said his mum smokes and he said he’s going to grow up and smoke and drink, he doesn’t care if he’s 13, he’s still going to do it”* (Female, Niuean, 10 years); *“Parents doesn’t even know younger people like some of our age sometimes go hide and smoke”* (Female, Tongan, 10 years).

## Discussion

When children encounter new objects and experiences, they look for information they can use to confirm, add to, or change their ideas [27]. When presented with a new behaviour a child first tries to understand the new object matching it against their existing knowledge. If the new object does not fit with what the child already knows, the child will try to come up with ways of understanding it or in some cases they classify it using a close-enough category [27]. Piaget called this ‘*classification’* that is, that children often group objects together on the basis of common features [27]. The rate at which a child can decipher and understand new experiences and objects also depends on the cognitive development of the child.

In the absence of prior knowledge of the electronic cigarette or inhaler, children in this study at first glance, classified the electronic cigarette and nicotine inhaler as a tobacco cigarette. Despite the commonalities, abnormal behavioural cues associated with the use of the electronic cigarette left some children with doubt about their classification. Thus, in an environmental context where the electronic cigarette or inhaler is new or rarely used, as in NZ currently, there is a risk that children will misperceive it as a tobacco cigarette and therefore as smoking. This could, as some opponents of cigarette-like nicotine delivery systems have feared, undermine de-normalisation strategies.

To mitigate the risk of misperception of the electronic cigarette or inhaler occurring, parents or family members that use these products could explain to children under their influence what the product is and why they are using it, particularly if they are using the product to reduce smoking-related harm. In our study, once the children were told about the novel devices and their potential use as cessation aids the children were pragmatic about the usefulness of the products for helping people stop smoking. Some children were also wary that use of these products would prohibit people from stopping smoking, because they were still going through the behavioral motions associated with smoking tobacco and they were still receiving nicotine. The children therefore, even at their age, could discern and express some of the competing thoughts that tobacco control advocates are grappling with: will cigarette-like nicotine delivery devices assist with reducing overall smoking prevalence or will they hinder that goal?

Children’s opinions on controversial topics such as smoking are important as they are affected at a similar or more severe rate than adults however they are usually unheard [1]. There have been several studies on children’s voices about cigarette smoking [1,28,29]. Participants in this study expressed concern for people who smoke or are exposed to smoke and they said smokers should quit for their own health. Glover and colleagues reported in their study that Māori and Pacific adult smoker’s reasons for quitting was for their own health, their family and children’s health [30]. Two key strategies to denormalise smoking behavior is for parents to quit smoking and educate their children about the cessation products they are using, so as to normalise quitting behavior.

The exploratory inductive nature of the enquiry was used to illicit children’s perceptions of the electronic cigarette and nicotine inhaler. Although our study did not test social learning theory in regards to children’s uptake of smoking in adulthood, we have learnt that if children are in an environment or social context where tobacco smoking is exclusively modelled by an adult children’s corresponding thoughts and views about the electronic cigarette and nicotine inhaler will be that they misperceive them as tobacco cigarettes. To mitigate the risk and to de-normalise smoking, education and raising awareness of these novel products is desirable.

### Study strengths

The strengths of this study is its originality. This is one of the first studies to be conducted to gain children’s perceptions about electronic cigarettes and nicotine inhalers.

### Limitations

The findings from the research were from a small sample size and participants were recruited from East and South Auckland thus limiting generalisabiliy to all Māori and Pacific children aged 6–10 years in NZ or to children of other ethnicities and countries. When individually interviewed, some children were reserved and said little. Thus it is hard to gauge if saturation was reached. Focus groups were useful for achieving fuller participation from more of the children. Conducting age-specific or gender-specific focus groups has been reported to facilitate discussion and provoke responses beyond those achieved through interviews [25,31].

Another limitation is that the children watched video clips and thus did not have the added cue of smell to alert them that the actor was perhaps not smoking a tobacco cigarette. However, even the sight of adults smoking has been shown to play an important role in uptake and normalising smoking. A case in point is the immense amount of research on smoking in movies where it has been found that increased exposure to smoking in movies has been associated with uptake [32]. Furthermore, we were also interested in children's perceptions at a distance at which they wouldn't necessarily be able to smell cigarette smoke. Inhaler use is also idiosyncratic in that some users may hold the inhaler and put it to their mouth as they would a tobacco cigarette and others do not. Our actor held the inhaler between thumb and forefinger which was different from how she smoked the tobacco cigarette. Thus it was a potential cue that they were not smoking a tobacco cigarette.

## Conclusions

The way children behave in the future directly links to adult’s role modelling behavior at present. Despite the electronic cigarette and nicotine inhalers function to aid smokers to quit some children in this study, at first glance did not discern the difference between the use of them and the smoking of a tobacco cigarette. Other children did notice anomalies in the ‘smoking’ behavior but without knowledge of the product they appeared to revert to their knowledge of cigarette smoking. To mitigate the risk of misperception, adult users could minimize use of cigarette-like devices in front of children or at the very least explain what the device is and that they are using it to help them stop smoking.

### Ethics approval

This project was conducted with the approval of the University of Auckland Human Participants Ethics Committee (ref: 2010/562).

## Competing interests

The authors declare that they have no competing interests.

## Authors’ contributions

JF recruited the participants, facilitated the focus groups and wrote the first draft. MG supervised JF, helped design the study and revised subsequent drafts. VN also supervised JF and was involved in the design of the study. FP revised subsequent drafts. All authors read and approved the final manuscript.
